# A Pharmacokinetic Bioequivalence Study Comparing Different‐Strength and ‐Size Capsules of Isavuconazonium Sulfate in Healthy Japanese Subjects

**DOI:** 10.1002/cpdd.1101

**Published:** 2022-04-11

**Authors:** Shinichiro Shirae, Yoko Mori, Tomohito Kozaki, Atsushi Ose, Setsuo Hasegawa

**Affiliations:** ^1^ Development Planning, Clinical Development Center Asahi Kasei Pharma Corporation Chiyoda‐ku Tokyo Japan; ^2^ Pharmaspur Inc. Tokyo Japan

**Keywords:** bioequivalence, isavuconazole, isavuconazonium sulfate, pharmacokinetics, safety

## Abstract

Isavuconazonium sulfate is the water‐soluble prodrug of the novel, broad‐spectrum, triazole antifungal agent isavuconazole. A size 0 elongated hard capsule containing 100 mg equivalent of isavuconazole is the currently marketed oral formulation in countries where it is approved. An alternative oral formulation, based on a lower‐strength and smaller‐size capsule, is required for pediatric and adolescent patients, as well as for some adult Japanese patients, especially those with difficulties swallowing larger capsules. This study was conducted to evaluate the bioequivalence of a size 0 elongated capsule containing 100 mg equivalent of isavuconazole and a size 3 capsule containing 40 mg equivalent of isavuconazole, after administration of 200 mg equivalent of isavuconazole (5 size 3 capsules or 2 size 0 elongated capsules) under fasted conditions. Bioequivalence of isavuconazole between the formulations was demonstrated, since point estimates (90%CI) for the ratio of the size 0 elongated capsules vs the size 3 capsules for maximum plasma concentration and area under the plasma concentration–time curve from time 0 to the last quantifiable concentration were within the acceptable range of 0.8 to 1.25. It was confirmed that both formulations were well tolerated, and no new safety signals were observed in healthy Japanese adult male subjects.

Isavuconazonium sulfate (BAL8557) is a water‐soluble triazole antifungal agent that is rapidly hydrolyzed by esterases to the active moiety, isavuconazole (BAL4815).^1^ Isavuconazole inhibits lanosterol 14α‐demethylase, a microsomal cytochrome P450 (CYP) enzyme essential for ergosterol biosynthesis in fungi.^2^ Isavuconazole has broad in vitro activity against a wide range of fungi.^3^ The dosage regimen is 200 mg equivalent of isavuconazole (corresponding to 372.6 mg of isavuconazonium sulfate) every 8 hours for 6 doses (48 hours), then once daily. With this dosage regimen, isavuconazonium sulfate demonstrated comparable efficacy to voriconazole.^3^, ^4^

Isavuconazole is metabolized mainly by CYP3A4 and CYP3A5, and further metabolized by uridine diphosphate glucuronosyltransferase (UGT).^5^ Following a single oral dose of cyano‐^14^C‐labeled isavuconazonium sulfate, ≈46.1% and 45.5% of the total sample radioactivity were recovered in feces and urine, respectively. Urinary recovery of unchanged isavuconazole was <1%.^1^, ^6^

Since isavuconazole is a sensitive substrate of CYP3A4, clinically significant impact on isavuonazole pharmacokinetics (PK) by concomitant use of strong CYP3A4 inhibitors or inducers have been reported.^5^, ^7^ As a result, concomitant use of strong CYP3A4 inhibitors such as ketoconazole and high‐dose ritonavir as well as strong CYP3A4 inducers such as long‐acting barbiturates, St John's wort, carbamazepine, and rifampin is contraindicated.^5^, ^7^, ^8^

Isavuconazole is a moderate CYP3A4 inhibitor and is reported to increase the systemic exposure of sensitive CYP3A4 substrates, such as midazolam, sirolimus, tacrolimus, cyclosporine, and atorvastatin.^5^, ^9^, ^10^ Additionally, isavuconazole is a mild inhibitor of UGT, P‐glycoprotein (P‐gp), and organic cation transporter 2 (OCT2), and isavuconazole has shown to increase the systemic exposure of UGT, P‐gp, and OCT2 substrate drugs, such as mycophenolate mofetil, digoxin, and metformin.^9^, ^10^ No clinically meaningful drug‐drug interactions have been reported for substrates of other CYP enzymes and another transporter, breast cancer resistance protein.^10^, ^11^, ^12^

The impact on isavuconazole PK by patient factors, such as age, sex, hepatic impairment, renal impairment, and race, have also been investigated. Among the factors investigated, no PK differences have been found to the extent of requiring dose adjustment.^13^, ^14^, ^15^, ^16^, ^17^, ^18^

Two formulations, intravenous and oral, are available for isavuconazonium sulfate. The oral formulation has a bioavailability of 98% and bioequivalence (BE) was demonstrated between the 2 routes of administration.^19^, ^20^ Therefore, the formulations can be switched without dose adjustment.^20^

Isavuconazonium sulfate has been used for the treatment of invasive aspergillosis and invasive mucormycosis in the United States and European countries.^21^ Clinical development of isavuconazonium sulfate for pediatric and adolescent patients is now being conducted.^22^ Additionally, a phase 3 study of isavuconazonium sulfate in Japanese patients has been conducted to support its potential approval for deep mycosis in Japan (ClinicalTrials.gov Identifier: NCT03471988).

A size 0 elongated hard capsule containing 100 mg equivalent of isavuconazole (corresponding to 186.3 mg of isavuconazonium sulfate) as an oral formulation has been used for adult subjects and patients in clinical trials of isavuconazonium sulfate, and it is the currently marketed formulation in countries where it is approved. A size 3 hard capsule containing 40 mg equivalent of isavuconazole (corresponding to 74.5 mg of isavuconazonium sulfate), a lower‐strength and smaller‐size capsule, has been investigated as a new oral formulation. This lower strength and smaller size of capsule is expected to be more appropriate for pediatric and adolescent patients and allows dose adjustment according to body weight.

Furthermore, a smaller‐size capsule may potentially lead to better patient acceptance and higher compliance, especially in pediatric and adolescent patients, where a larger size of tablets and/or capsules may cause difficulty swallowing. Difficulty swallowing can lead to a variety of adverse events (eg, ulceration, stricture, perforation, pain, gagging, choking, and aspiration) and patient noncompliance with treatment regimens.^23^, ^24^, ^25^, ^26^

A size 3 capsule of isavuconazonium sulfate may also be suitable for many adult Japanese patients with deep mycosis, especially those with dysphagia and other difficulties with swallowing larger capsules. Interviews of Japanese health care professionals showed that a size 3 capsule is preferable to a size 0 elongated capsule in Japanese clinical settings to avoid difficulty swallowing and then to improve safety and compliance. For these reasons, a size 3 hard capsule containing 40 mg equivalent of isavuconazole (corresponding to 74.5 mg of isavuconazonium sulfate) is being developed for potential use in Japan as an alternative oral formulation.

This phase 1 study was designed to assess the BE of a single oral dose of 200 mg equivalent of isavuconazole (corresponding to 372.6 mg of isavuconazonium sulfate) administered as 2 size 0 elongated capsules or 5 size 3 capsules in healthy Japanese adult male subjects.

## Methods

### Study Design

This study was conducted in accordance with the International Conference on Harmonisation E6 Good Clinical Practice. The study was performed at Clinical Research Hospital Tokyo, and the study protocol was reviewed and approved by an institutional review board (IHL Shinagawa East One Medical Clinic Institutional Review Board, Tokyo, Japan). Written, informed consent was obtained from each subject before any study‐related procedures were performed. This research was compliant with the principles expressed in the Declaration of Helsinki.

This was a single‐center, randomized, open‐label, single‐dose, 2‐treatment and 2‐period crossover phase 1 study in healthy Japanese male subjects. A total of 70 healthy Japanese male subjects were randomly assigned to 2 groups of 35 subjects each. Subjects were administered a single dose of 200 mg equivalent of isavuconazole (corresponding to 372.6 mg of isavuconazonium sulfate) as 2 capsules of size 0 elongated capsules (formulation A) or as 5 capsules of size 3 capsules (formulation B) in a formulation A‐B (group A) or B‐A (group B) sequence under the fasted condition with a 35‐day washout period between each treatment period.

### Study Population

Healthy Japanese male volunteers aged 20 to 44 years who were capable of sufficiently comprehending a description of the study and competent to give informed consent were recruited. Healthy subjects were defined as having no clinically relevant abnormalities as identified by medical history, physical examination, vital signs, 12‐lead electrocardiogram (ECG), and clinical laboratory tests. Key exclusion criteria included body weight <50.0 kg; body mass index <18.5 or >25.0 kg/m^2^; and evidence of liver disease or liver injury as indicated by abnormal liver test results, such as alanine aminotransferase, aspartate aminotransferase, or γ‐glutamyl transferase levels exceeding the upper limits of normal. Subjects with a history of short QT syndrome or Fridericia corrected QT interval <360 milliseconds were also excluded because dose‐related shortening of the corrected QT interval was reported, and isavuconazonium sulfate is contraindicated in patients with familial short QT syndrome.^27^

### Bioanalytical Methods for PK Samples

Concentrations of isavuconazole in plasma were measured using a validated liquid chromatography coupled with tandem mass spectrometry method.^28^ Isavuconazole levels were measured in all plasma samples taken from subjects who received isavuconazonium sulfate. The lower limit of quantification was 5 ng/mL.

### Pharmacokinetic Assessments and Statistical Analyses

Plasma samples for the measurement of isavuconazole concentration were taken at the following time points: before dosing; at 15 and 30 minutes; and 1, 1.5, 2, 2.5, 3, 3.5, 4, 4.5, 5, 6, 10, 24, 48, 72, 96, 120, 192, 288, 432, and 624 hours after isavuconazonium sulfate administration. After collection, samples were processed immediately and stored at –80°C until shipment to the central research laboratory.

PK parameters were calculated by noncompartmental analysis using WinNonlin Version 7.0 (Pharsight Corporation, Saint Louis, Missouri). Calculations were based on the actual sampling times recorded during the study. The isavuconazole PK parameters, including maximum plasma concentration (C_max_), area under the plasma concentration–time curve (AUC) from time 0 to the last quantifiable concentration (AUC_last_), AUC from time 0 to infinity (AUC_inf_), time to C_max_, and terminal elimination half‐life, were determined. The PK analysis set included all subjects who received at least 1 dose of isavuconazole and had the plasma concentration measurement results required for the PK analyses.

After logarithmic transformation, C_max_ and AUC_last_ of treatment A or treatment B were analyzed separately using mixed‐effects model fitting for sequence, formulation, and period as fixed effects and subject nested in sequences as a random effect. Point estimates and associated 90%CIs for the difference in means of the 2 treatments were constructed. These estimated values were exponentially back‐transformed to provide point estimates and associated 90%CIs for the geometric mean ratio. The treatments were considered to be bioequivalent if the 90%CIs of the geometric mean ratio were within the acceptable range of 0.8 to 1.25. The analysis set for BE analyses (BE analysis set) included all subjects in the PK analysis set who had PK parameters for both formulations.

The sample size was calculated on the basis of the estimated intrasubject variability and the difference in means of the 2 formulations. The intrasubject variability was estimated as 19.4 from a previously conducted PK study in healthy Chinese volunteers, and the difference in means of the 2 formulations was estimated as 1.15. A sample size of 68 subjects was estimated as able to meet BE criteria with 80% power. A total of 70 subjects were enrolled to ensure the completion of at least 68 subjects.

### Safety Assessment

Safety assessments included physical examination, vital signs (blood pressure, heart rate, and axillary temperature), 12‐lead ECG, laboratory tests (hematology, biochemistry, and urinalysis), and adverse events. The safety analysis set included all subjects who received at least 1 dose of isavuconazonium sulfate.

## Results

### Subject Disposition and Demographics

A total of 70 healthy Japanese male subjects were enrolled in the study. All 70 subjects received at least 1 dose of isavuconazonium sulfate, and 66 subjects completed the study. Three subjects, 2 subjects in group A and 1 subject in group B, were withdrawn from the study in period 1, and 1 subject in group B was withdrawn from the study in period 2. A total of 68 subjects received the size 0 elongated capsules, and 69 subjects received the size 3 capsules.

A total of 68 subjects and 69 subjects were included in the PK analysis set for the size 0 elongated capsules and the size 3 capsules, respectively. A total of 66 subjects were included in the BE analysis set.

Demographics and baseline characteristics of the BE analysis set are summarized in Table 1. No appreciable differences in any baseline characteristics were observed between the groups.

**Table 1:** Summary of Demographic Characteristics

	N	Age, y	Height, cm	Weight, kg	BMI, kg/m^2^
Group A	33	29.8 ± 7.1	169.6 ± 6.7	62.1 ± 7.6	21.6 ± 2.0
Group B	33	28.0 ± 7.3	171.0 ± 4.7	61.9 ± 5.7	21.1 ± 1.5

Values are presented as arithmetic means ± standard deviation.

Group A: size 0 elongated capsule–size 3 capsule sequence.

Group B: size 3 capsule sequence–size 0 elongated capsule.

### Pharmacokinetics

**Figure 1:**
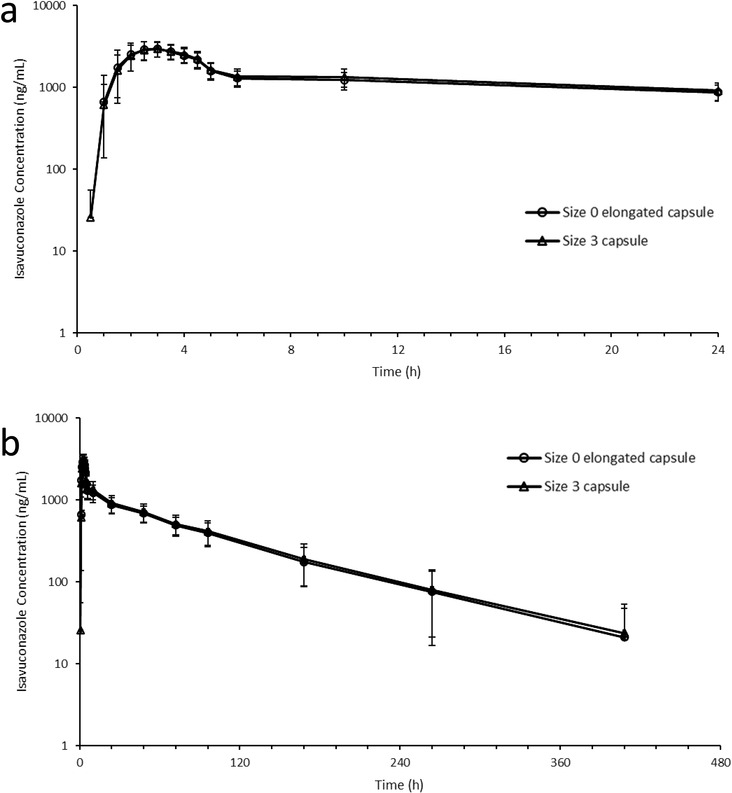
Mean and standard deviation of the plasma isavuconazole concentration–time profile after single administration of 200 mg equivalent of isavuconazole by size 0 elongated capsule or size 3 capsule. (a, b): mean plasma concentration–time profile after single oral administration of isavuconazonium sulfate at 24 hours (a) and up to 480 hours (b).

The mean (± standard deviation) plasma concentration–time profiles of isavuconazole after administration of 200 mg equivalent of isavuconazole (corresponding to 372.6 mg of isavuconazonium sulfate) as 2 capsules of the size 0 elongated capsules or as 5 capsules of the size 3 capsules are presented in Figure 1. The plasma concentration–time profiles of isavuconazole of the 2 formulations were similar.

PK parameters of isavuconazole after each treatment are presented in Table 2. Similar values were seen in the PK parameters between the formulations. The 90%CIs for C_max_ and AUC_last_ were within the acceptable range (0.8‐1.25).

**Table 2:** Summary of Pharmacokinetic Parameters and Mean Difference of Size 0 Elongated Capsule or Size 3 Capsule After a Single Oral Administration of 200 mg Equivalent of Isavuconazole

	Size 0 Elongated Capsule	Size 3 Capsule	Ratio^a^	90%CI
N	66	66		
C_max_, ng/mL	3296 ± 614	3277 ± 576	0.996	0.953‐1.040
AUC_last_, ng × h/mL	112229 ± 30289	117913 ± 33142	1.047	1.015‐1.080
AUC_inf_, ng × h/mL	113607 ± 30728	120143 ± 33274	1.054	1.024‐1.085
t_1/2_, h	69.4 ± 28.7	69.5 ± 27.8	NC	NC

AUC_inf_, area under the plasma concentration–time curve from time 0 to infinity; AUC_last_, area under the plasma concentration–time curve from time 0 to the last quantifiable concentration; C_max_, maximum plasma concentration; NC, not calculated; t_1/2_, terminal elimination half‐life.

Pharmacokinetic parameters are summarized for the analysis set for bioequivalence analyses.

Values are presented as arithmetic means ± standard deviation.

^a^ Ratio: Geometric mean ratio of size 0 elongated capsule/size 3 capsule.

### Safety

There were no serious adverse events or severe treatment‐emergent adverse events (TEAEs). One subject was withdrawn from the study due to TEAEs after administration of the size 3 capsules. The TEAEs were headache, nausea, and vomiting, which were assessed as drug‐related TEAEs. The headache was mild, and nausea and vomiting were moderate in severity. These TEAEs resolved without concomitant treatment.

TEAEs were reported in 16 of 68 subjects after administration of the size 0 elongated capsules and 24 of 69 subjects after administration of the size 3 capsules. Drug‐related TEAEs were reported in 3 subjects after administration of the size 0 elongated capsules and 2 subjects after administration of the size 3 capsules. All TEAEs were mild or moderate in severity. There were no clinically significant findings in vital signs, body weight, and 12‐lead ECG.

## Discussion

This phase 1 study was conducted to assess the BE of a single oral dose of 200 mg equivalent of isavuconazole (corresponding to 372.6 mg of isavuconazonium sulfate) administered as size 0 elongated capsules and size 3 capsules in healthy Japanese adult male subjects. A new formulation, based on a lower‐strength and smaller‐size capsule product, has been developed with the aim of more flexibility for dose adjustment according to body weight and of improvement in safety and compliance in pediatric and adolescent patients. This new, smaller capsule also has a high likelihood of contributing to safety and compliance in many Japanese patients with deep mycosis, especially those with dysphagia and other difficulties with swallowing larger capsules.

The plasma concentration–time profile and PK parameters were similar for the treatments. The mean pharmacokinetic parameters in the present study were similar to the ones previously reported in healthy adult Japanese subjects after single oral administration of 200 mg equivalent of isavuconazole (C_max_, 2933 ng/mL; AUC_last,_ 107 802 ng × h/mL; AUC_inf_, 110 737 ng × h/mL).^29^ For the ratio of the size 0 elongated capsules versus size 3 capsules, the 90%CIs for C_max_ and AUC_last_ were within the acceptable range of 0.8 to 1.25. Therefore, BE was demonstrated between 200 mg equivalent of isavuconazole administered as size 0 elongated capsules and size 3 capsules.

It was confirmed that a single oral dose of 200 mg equivalent of isavuconazole administered as size 0 elongated capsules and size 3 capsules was well tolerated, and no new safety signals were observed in healthy Japanese adult male subjects.

Overall, this study demonstrated that size 3 capsules of isavuconazonium sulfate are safe and bioequivalent with size 0 elongated capsules when administering 200 mg equivalent of isavuconazole. The lower‐strength and smaller size 3 capsules are thought to be more appropriate for pediatric and adolescent patients in terms of flexible dose adjustment by body weight, adverse events, and compliance due to difficulty swallowing compared to the size 0 capsule. Although the efficacy and safety of isavuconazonium sulfate need to be confirmed in clinical studies in pediatric and adolescent patients, providing the option of the size 3 capsules may provide a new and better treatment option for patients in this subgroup with deep mycosis.

This smaller‐size capsule also likely reduces the occurrence of adverse events associated with swallowing difficulties and compliance in many Japanese patients with deep mycosis, especially those with dysphagia and other difficulties with swallowing larger capsules. Furthermore, to avoid difficulty swallowing and then to improve safety and compliance, a size 3 capsule is preferable to a size 0 elongated capsule in Japanese clinical settings based on our interviews of Japanese health care professionals. Therefore, providing the option of the size 3 capsule should help achieve the proper oral administration of isavuconazonium sulfate in Japan.

## Conclusion

BE was demonstrated between size 0 elongated and size 3 capsules of isavuconazonium sulfate when administering 200 mg equivalent of isavuconazole (corresponding to 372.6 mg of isavuconazonium sulfate). It was confirmed that a single oral dose of 200 mg equivalent of isavuconazole administered as size 0 elongated capsules and size 3 capsules was well tolerated, and no new safety signals were observed in healthy Japanese adult male subjects.

## Conflicts of Interest

S.S., T.K., and A.O. are employees of Asahi Kasei Pharma. Y.M. was an employee of Asahi Kasei Pharma at the time of the study conduct. S.H. received a consulting fee from Asahi Kasei Pharma. This work was funded by Asahi Kasei Pharma Corporation.
